# Urinary Extracellular Vesicles and Salt-Losing Tubulopathies: A Proteomic Approach

**DOI:** 10.3390/proteomes8020009

**Published:** 2020-05-09

**Authors:** Francesca Raimondo, Clizia Chinello, Luigi Porcaro, Fulvio Magni, Marina Pitto

**Affiliations:** 1 School of Medicine and Surgery, University Milan Bicocca, 20900 Monza, Italy; clizia.chinello@unimib.it (C.C.); fulvio.magni@unimib.it (F.M.); marina.pitto@unimib.it (M.P.); 2 Laboratory of Medical Genetics, Fondazione IRCCS Ca’ Granda Hospital, 20162 Milan, Italy; luigi.porcaro@policlinico.mi.it

**Keywords:** exosomes, extracellular vesicles, Gitelman syndrome, Bartter syndrome, diagonal electrophoresis

## Abstract

Renal tubular cells release urinary extracellular vesicles (uEV) that are considered a promising source of molecular markers for renal dysfunction and injury. We investigated uEV proteomes of patients with hereditary salt-losing tubulopathies (SLTs), focusing on those caused by Gitelman and Bartter (BS) syndromes, to provide potential markers for differential diagnosis. Second morning urine was collected from patients with genetically proven SLTs and uEV were isolated by the ultracentrifugation-based protocol. The uEV proteome was run through a diagonal bidimensional electrophoresis (16BAC/SDS-PAGE), to improve hydrophobic protein resolution. Sixteen differential spots from the proteome of two variants (BS2 and BS3) were analysed by nLC-ESI-MS/MS after in-gel tryptic digestion. A total of 167 protein species were identified from 7 BS2 spots and 9 BS3 spot. Most of these proteins were membrane-associated proteins, in particular transmembrane proteins, and were related to typical renal functions. The differential content of some uEV was then validated by immunoblotting. Our work suggests that uEV proteomics represents a promising strategy for the identification of differential SLT proteins. This could play a role in understanding the pathophysiological disease mechanisms and may support the recognition of different syndromes.

## 1. Introduction

Gitelman syndrome (GS) and Bartter syndrome (BS) are rare inherited salt-losing tubulopathies (SLT) characterized by hypokalaemic, hypochloraemic metabolic alkalosis [1,2]. They are marked by lower sodium chloride reabsorption of the distal nephron, which results in extracellular volume contraction and increased activity of the renin angiotensin aldosterone system [3]. They are caused by mutations in at least seven genes, involved in sodium/fluid reabsorption, in the thick ascending limb of the loop of Henle and/or the distal convoluted tubule [2,4]. Different classifications have been proposed based on clinical symptoms and/or on the underlying genetic cause. Currently, genetic testing is the main diagnostic tool in use for all known genes responsible for GS and BS, however it is expensive and time-consuming. Moreover, in some patients, genetic screening is not readily available or disease-attributable mutations cannot be found [5]. In addition, a poor phenotype-genotype relationship has been reported. This is likely due to the interaction with other cotransporters and different degrees of compensation through alternative pathways [6].

In particular, given their phenotypic (i.e., clinical and biochemical) similarity, it is sometimes challenging differentiate between GS and BS type 3 (BS3) and between BS type 1 (BS1) and BS Type 2 (BS2). GS and BS3 may be clinically indistinguishable, especially in the absence of a certain genetic diagnosis [7,8]. Furthermore, cases of BS2 have been reported with milder phenotypes resembling BS1 [9,10]. Such diagnostic ambiguity can lead to inappropriate treatment choices and ultimately affect patients’ health [11].

In a previously published paper [12], we showed the diagnostic potential of urinary extracellular vesicles (uEV) in the recognition of SLT. In fact, the molecular composition of this nanosized particles is reported to depend upon the type, and even status, of the producer cell [13]: uEV membranes contain all of the major renal transport proteins belonging to apical and basolateral proteins from all nephron segments, and are believed to reflect their activity in the kidney [14]. In particular, it was shown that it is possible to discriminate GS and BS1 patients from other SLT, based on differential abundance of the thiazide-sensitive sodium chloride cotransporter (NCC) and of the furosemide-sensitive sodium-potassium-chloride cotransporter (NKCC2) within uEV of patients, by western blotting [12,15].

This work highlights further differences in membrane protein composition among uEV from patients affected by tubulopathies, using an unconventional proteomic analysis. In fact, we applied a proteomic approach based on diagonal bi-dimensional electrophoresis (16BAC/SDS-PAGE), able to improve hydrophobic protein resolution, followed by LC-ESI-MS/MS analysis of some selected differential spots, after in-gel tryptic digestion. Finally, we proposed a preliminary panel of proteins whose differential content in patient uEV may allow discrimination among the different SLT.

## 2. Materials and Methods

### 2.1. Materials

Milli-Q water was used for all solutions. Benzyldimethyl-n-hexadecylammonium Chloride (16-BAC), Bicinchoninic acid (BCA) protein assay, acetic acid, isopropyl alcohol, methanol, bovine serum albumin (BSA), *N*-cyclohexyl-3-aminopropanesulfonic acid (CAPS), ammonium bicarbonate (NH_4_HCO_3_), dithiothreitol (DTT), iodoacetamide (IAA), acetonitrile (ACN), formic acid (FA) and pyronin-Y were supplied from SIGMA Chemical Co. (St. Louis, MO, USA); glycerol was from Merck (Darmstadt, Germany). Coomassie Blue R205 and urea were from Bio-Rad (California, USA). Hybond-enhanced chemiluminescence (ECL) nitrocellulose membrane was from GE (Little Chalfont, Buckinghamshire, UK). NuPAGE^®^ SDS-PAGE Gel Electrophoresis System components (mini-gels, running and loading buffer, molecular weight markers and gel protein stainings) were supplied by Life Technologies (Paisley, Renfrewshire, UK). Protease inhibitor cocktail (Complete) was from Roche (Monza, Italy). Anti-NCC- AB118003, anti-annexin A2 (ANXA2), code AB41803, anti-carbonic anhydrase 2 (CA2), code AB382559 polyclonal antibodies (pAb), anti-vacuolar-type ATPase B subunit 1 (VATB1), clone 3B11, code AB118003, anti-sodium/proton exchange regulatory cofactor NHE-RF1 (NHERF1), code AB88238, anti-Tumour Susceptibility Gene 101 (TSG101) and anti-Motility-related protein 1 (CD9) monoclonal antibodies were from Abcam (Cambridge, UK); anti-NKCC2, code HPA018107, pAb was from SIGMA Chemical Co. (St. Louis, MO, USA). Anti-Ammonium transporter Rh type C (RHCG), code H00051458-M06, and anti-GAIP interacting protein, C terminus (GIPC2) mAb were from NovusBio (Centennial, CO, USA). Anti-Flotillin 1 (FLOT1) mAb was purchased from Transduction Laboratories (Lexington, KY, USA) and anti-aquaporin 2 (AQP2) mAb from Cell Signaling Technology (Beverly, MA, USA). Species-specific secondary peroxidase conjugated antibodies and ECL reagents were from Pierce (Rockford, IL, USA).

### 2.2. Patients

The patients included in this study are from the same cohort already characterized in [12]: Table 1 shows the cases that were selected for the proteome analysis reported in the present paper (Table 1).

The patients were studied and followed up at San Leopoldo Mandic Hospital (Merate, Italy). They all had normotensive and chronic ionic disturbances of tubular/uncertain origin. Laboratory tests displayed alterations suggestive for SLTs, such as hypokalaemia, hypo-/hypercalciuria, normo-/hypomagnesaemia and metabolic alkalosis. No proteinuria was detected in the collected samples. Molecular diagnosis, performed by the Laboratory of Medical Genetics of Fondazione IRCCS Ca’ Granda Maggiore Policlinico Hospital (Milan), disclosed significant mutations of pertinent genes in the homozygous or compound heterozygous state (Table 1).

### 2.3. Urine Collection and Exosome Isolation

Urine collection, uEV isolation and characterization were performed as described [12]. Briefly, second morning urine samples (~30–50 mL) were collected according to the Human Kidney and Urine Proteome Project guidelines (HKUPP, http:www.hkupp.org). After sediment removal (10 min at 1.000 g, 4 °C), anti-proteases were added to the urine (Complete, Roche) and stored at −80 °C. An aliquot of the collected urine samples was submitted to routine chemical-physical examination and creatinine assay (Jaffé method, Roche) [12].

uEV were prepared from the stored urine samples by differential centrifugation [12,16]. Briefly, thawed urines were centrifuged at 17.000× *g* for 15 min and at 200.000× *g* for 70 min: crude uEV pellets were washed in phosphate buffered saline (PBS) solution (200.000× *g* for 70 min), resuspended in PBS containing protease inhibitors and stored at −80 °C until use.

### 2.4. Electrophoresis and Western Blotting Analysis

#### 2.4.1. Benzyldimethyl-n-Hexadecylammonium Chloride/Sodium Dodecyl Sulfate Polyacrylamide Gel Electrophoresis (16-BAC/SDS-PAGE)

The 16-BAC/SDS-PAGE protocol was performed according to [17]. Briefly, we prepared 7.5% separation and 4% stacking gel using 1-mm spaced glass plates (ATTO Corporation, Tokio, Japan). uEV samples were mixed with 16-BAC /PAGE 2X loading buffer (7.5 M urea, 250 mM 16-BAC, 10% glycerol, 75 mM DTT, 5% pyronin-Y), heated at 60 °C for 5 minutes, and loaded into wells. Electrophoresis was conducted in acid electrode buffer (2.5 mM 16-BAC, 150 mM glycine, and 50 mM phosphoric acid) at 20 mA/gel. After the separation, uEV proteins were fixed in isopropyl alcohol /acetic acid/H_2_O solution (3.5/1/5.5 *v*/*v*) by changing the solution every 10 minutes and this was performed for a total of 6 times. Subsequently, the gel was stained in Coomassie Blue R205 (0.15% in fix solution) overnight whilst being agitated. After destaining with fix solution, gel was equilibrated in 100 mM Tris-HCl (pH 6.8), three washing for ten minutes each. Sample lanes were excised and incubated with NuPAGE loading buffer 3X/150 mM DTT, for 5 minutes, before loading onto 1 mm 4–12% NuPAGE gels (IPG well) for second dimension separation (NuPAGE Electrophoresis System, Life Technologies, Paisley, Renfrewshire, UK). Proteins were separated in MOPS SDS-Running buffer and were stained by SYPRO™ Ruby Protein Gel Stain (for analytical gels, loaded with 20 µg of uEV proteins) or SimplyBlue Safe Stain (for preparative gels, loaded with 40 µg of uEV proteins) (Life Technologies), following manufacturer’s instructions. In gels to be used for spot excision, pools of uEV from 3 or 4 individuals for each condition were used, mixing equal amount of proteins for each subject. This was done in order to minimize the effect of biologic variability and to obtain a clear visualization on the gels. Gel images were acquired by a CCD camera equipped with a UV or white light transilluminator (LAS4000, GE).

#### 2.4.2. Western Blotting Analysis

Equal amount of uEV proteins were separated using 4−12% NuPAGE in MOPS SDS buffer (Life Technologies), as described [12,16], and transferred to nitrocellulose membranes using a “tank” electrophoretic transfer apparatus (Hoefer) in CAPS transfer buffer (10 mM CAPS pH 11, 10% Methanol). The filters were saturated with 5% free-fat milk/0.2% Tween 20 in PBS solution, and then incubated with the primary antibodies followed by peroxidase-conjugated secondary antibodies. Protein signals were developed using enhanced chemiluminescence detection (SuperSignal West-Dura/Femto ECL, Pierce) by the LAS4000 CCD camera (GE). Densitometric analysis was performed by ImageQuant TL software (GE Healthcare).

### 2.5. Mass Spectrometry and Protein Identification

All the spots were excised and stored at 4 °C. The spots of interest were subjected to in-gel protein digestion as already described ([16] I. Briefly, the gel slices were washed twice with a mixture of 25 mM NH_4_HCO_3_/ACN (1:1; *v*/*v*) for 15 min. Proteins were reduced by 10 mM DTT, 45 min at 56 °C, and then alkylated by 55 mM IAA, 30 min in dark. Gel slices were then washed using 25 mM NH_4_HCO_3_/50% acetonitrile for three times. After dehydration with ACN, the proteins were in-gel digested by modified porcine trypsin (Promega, Madison, WI, USA; 12.5 ng mL^−1^ in 25 mM NH4HCO3) at 37 °C overnight. The obtained peptide solutions were then diluted in 0.1% TFA and desalted using Ziptip™-C18 (Millipore, Burlington, MA, USA) following manufacturer’s instructions.

Protein identification was performed on a Dionex UltiMate 3000 rapid separation (RS) LC nanosystem (Thermo Scientific, Germany) coupled with Impact HDTM UHR-qToF system (Bruker Daltonics, Bremen, DE). After injection, peptide samples were desalted and concentrated onto a 2 cm precolumn (Dionex, Acclaim PepMap 100 C18, cartridge, 300 mm ID5 mm, 5 mm), and separated by a 200 min-multistep gradient (mobile phase 80% acetonitrile/0.08% FA) on the analytical 50 cm nanocolumn (Dionex, 0.075 mm ID, Acclaim PepMap100, C18, 2 mm) with a flow rate of 300 nl/min. The mass spectrometer was operated in DDA (Data Dependent Acquisition Mode), with automatic switching between full scan MS and MS/MS acquisition. N_2_ was used as a gas for CID (collision induced dissociation) fragmentation. The software automatically selected the number of precursor ions in order to fit into a fixed cycle time: the time between two subsequent MS acquisitions was 5 s. The charge of precursor ions ranged between +2 and +5, and precursor peaks above 1575 counts, in 300–1221 and 1225–2200 *m*/*z* window, were selected. IDAS (Intensity Dependent Acquisition Speed) and RT2 (RealTime Re-Think) functionalities were applied. A specific lock-mass (1221.9906 *m/z*) and a calibration segment of 10 mM sodium-formate cluster solution before the beginning of the gradient were applied in order to achieve an improvement of mass accuracy. The obtained chromatograms were elaborated by Compass DataAnalysisTM, v.4.0 Sp4 (Bruker Daltonics, Hamburg, Germany). The resulting mass lists were processed using an in-house Mascot search engine (v.2.4.0), through Mascot Daemon, considering the human Swissprot database (accessed May 2017, 555.594 total entries) as reference database. We set trypsin as enzyme and carbamidomethyl (C) as fixed modification in the search parameters. Mass tolerance for all identifications was generally fixed at 20 ppm for the precursor ions and 0.05 Da for the product ions. We applied automatic decoy database search and a built-in Percolator algorithm to calculate the probabilities of posterior error for each peptide-spectrum match and to rescore search results with a unique significance threshold. Global false discovery rate < 1% was considered to filter the data and we considered identified only proteins with at least one unique identical peptide sequence (*p*-value < 0.05) to be positively identified.

### 2.6. Bioinformatic Analysis

Gene ontology (GO) analysis was performed using UniprotKB (http://www.uniprot.org/uniprot/) database, considering the molecular function. The subcellular localization of the identified proteins was assigned by UniprotKB (http://www.uniprot.org/uniprot/), LocDB (www.rostlab.org/services/locDB/) and the Human Protein Atlas (www.proteinatlas.org) databases. Tissue specificity was evaluated comparing the lists of the identified proteins with the human tissue specific proteome, based on transcriptomics analysis provided by the Human Protein Atlas (www.proteinatlas.org) [18,19]. We also considered the “expression protein score” across all major organs and tissue types in the human body (www.proteinatlas.org).

### 2.7. Statistical Analysis

Differences in the abundance level of the uEV proteins obtained by WB were evaluated by the non-parametric Kruskal-Wallis test (two-sided, α = 0.05), coupled with a post-hoc Dunn’s multiple comparison. We targeted specific two by two comparisons by the non-parametric Mann-Whitney test (one-tail, α = 0.05). All the statistical analysis was performed by GraphPad Prism. We performed a multivariate analysis using the web tool ClustVis (https://biit.cs.ut.ee/clustvis/) [20]. We chose “correlation” to measure the clustering distance and “average” as linkage criterion.

## 3. Results

### 3.1. uEV Characterization

Proteomic analysis of uEV requires first to correctly characterize the vesicles. We prepared uEV from urine samples of patients enrolled in our previous study on SLT [12]. As shown in Supplementary Materials, Figure S1, uEV proteins display a typical electrophoretic profile, different from that of the urine of origin and the supernatant after ultracentrifugation. In particular, they show enrichment of uromodulin, the most abundant urinary glycoprotein, and depletion of albumin (Figure S1a). Samples of uEV of patients showed quite similar protein pattern in the different SLT and healthy subjects.

Moreover, uEV markers, such as FLOT1, TSG101, CD9 (ubiquitary exosomal markers) and AQP2 (specific for urine) [21] were highly, and rather uniformly, enriched in the vesicle fractions of the different subjects, while their signals were nearly undetectable in total urine and in the supernatant after ultracentrifugation (Figure S1b).

uEV prepared by the same protocol in our lab were characterized by electron microscopy and results were shown in a previous paper of our group [16]. Finally, the isolation protocol was further validated by nanoparticle tracking analysis (NTA) performed on uEV obtained from healthy subjects in the pediatric age group (data not shown), confirming that the size distribution is typical of uEV [22], with the peak of the most represented vesicle population positioned within the range of 101–200 nm in diameter.

### 3.2. Two-Dimensional 16-BAC/SDS Polyacrylamide Gel Electrophoresis Separation of uEV Proteins

**Figure 1:**
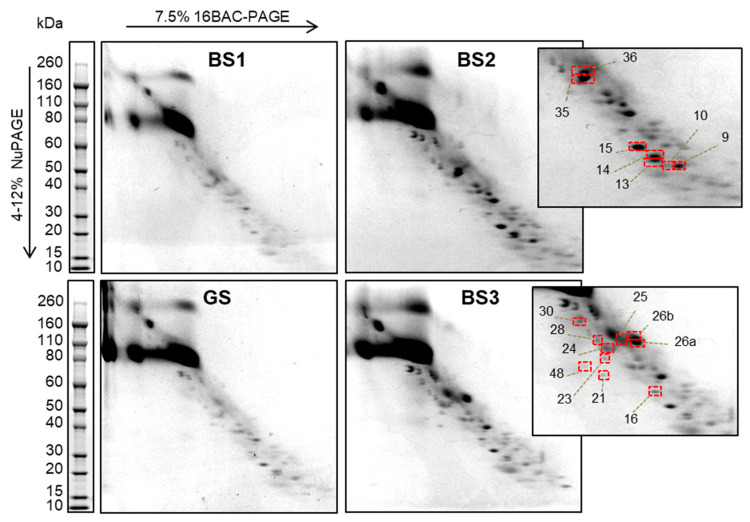
uEV protein profile by two-dimensional (2D) diagonal electrophoresis. Protein separation of uEV from Gitelman Syndrome (GS), Bartter type 1 (BS1), Bartter type 2 (BS2) and Bartter type 3 (BS3) patients by 16-BAC-PAGE/SDS-PAGE and Blue Coomassie staining. In the right panels, the selected spots from BS2 (upper) and BS3 (lower) are highlighted by red rectangles.

Two-Dimensional 16-BAC/SDS-PAGE requires a large amount of proteins (at least 40 µg for each sample), which it is hard to obtain from patient uEV, especially in a paediatric clinical setting (due to limited urine volume). Moreover, uEV contain only 2–3% of total urine proteins [23]. In addition, SLT patient urine is known to be quite diluted. The use of the highly sensitive protein staining, SyproRuby, allowed us to use half of the material (20 µg) to detect protein signals and to test the reproducibility of this electrophoretic technique. Examples are shown in Figure S2. For technical reproducibility, the same uEV sample was analysed twice, at different times, and gave similar profiles. Moreover, when analysing biological replicates (for three healthy patients) protein profiles were observed to be similar. Since those results were reproducible, we proceeded to analyse uEV pools from patients with the same SLT (see Figure 1). Moreover, pooling samples from biological replicates reduced biological variability and improved spot visualisation on the gel. The images of the gels show that most spots align along the diagonal, as expected. However, a few spots appear shifted from the diagonal. They likely represent hydrophobic proteins that show a different electrophoretic mobility in first and second dimension [17]. Since a critical point in the differential diagnosis of hereditary tubulopathies is the discrimination between BS1 and BS2 and between BS3 and GS, we selected spots by focusing on the comparison between protein profiles of uEV of the BS1 vs. BS2, and BS3 vs. GS pools (Figure S3). We picked 7 spots in the BS2 gel and 9 in the BS3 (Tables S2 and S3). Such selected spots—marked in red in fig 1 insert—were excised from gels of uEV pools BS2 and BS3 and were then analysed after in-gel tryptic digestion followed by nLC-ESI-MS/MS in order to identify the underlying protein species.

### 3.3. Identification of uEV Proteins

**Figure 2:**
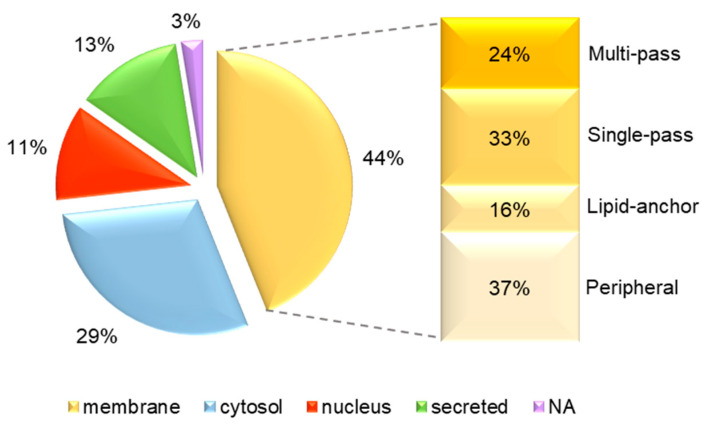
Subcellular localization of uEV proteins. The pie chart represents the cellular localization of the proteins identified in the 16-BAC/SDS-PAGE spots of BS2 and BS3 patient uEV. On the right, the percentages of integral (single-pass and multi-pass), lipid-anchor and peripheral membrane proteins are shown.

MS analysis identified 167 different protein species, 19 of which in both BS2 and BS3 uEV (Table 2 and Table S1). The lists of proteins identified in each spot are reported in Tables S2 and S3). Keratins were excluded as they are common contaminants in LC-MS/MS data. Moreover, we decided to not include peptides derived from uromodulin. Proteins that were recovered in more than one spot were counted only once. Even if only a few spots were analysed, we can consider them as roughly representative of the whole uEV proteomes separated by the diagonal electrophoresis, because they were chosen without pre-determined criteria except the differential intensity between two patient groups. The results show that the most represented subcellular location is membrane (44%) (Table S4): inside this group, the transmembrane proteins (single pass + multi pass) are the most abundant, presenting more than 55% (Figure 2). This suggests that the 16-BAC/SDS-PAGE is also able to enrich for membrane proteins in the case of uEV, which, to our knowledge, have never been analysed by this method.

**Figure 3:**
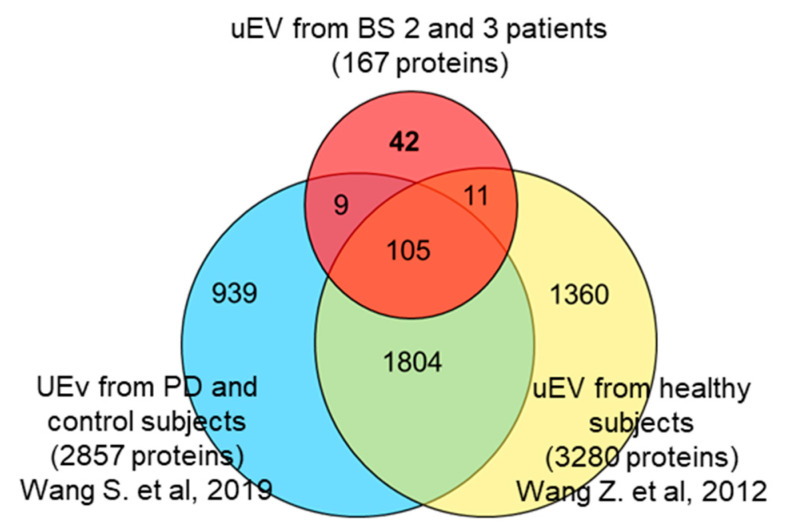
Venn diagram. Comparison of proteins identified in the partial proteome of uEV from BS2 and BS3 patients and the whole proteome of uEV from healthy subjects [24] and Parkinson’s disease patients and healthy controls [25].

It is worth noting that when comparing our protein list with the fullest set of healthy subject uEV proteins (3280 entries), published by Wang Z. et al. [24] and the more recent one by Wang S. et al. (2873 entries) [25], 42 proteins were not shared with those datasets (Figure 3), and were identified only in our samples (underlined in Table 2). Fourteen of them (33%) were membrane proteins, and interestingly, they are all integral transmembrane proteins (50% multi-pass and 43% single-pass) except for a peripheral one.

The list of identified proteins (Table 2 and Table S1) provides a preliminary reservoir for the detection of differences in the proteome of uEV among SLTs. Further analysis of these proteins might help elucidating the mechanisms of SLT physiopathology. Among the identified proteins, we could find many typical exosome proteins, such as those involved in biogenesis or structural processes. In particular, TSG101 (tumor susceptibility gene 101), Flotillin 1 (FLOT1) and the Vacuolar Protein Sorting-associated proteins 4 A and B were identified. Moreover, many typical renal proteins, such as Aquaporin-2 (AQP2), Dipeptidase-1 and Carbonic Anhydrase 2 (CA2), as well as some proteins belonging to the Annexin family, such as Annexin-A7 (ANXA7) and Annexin-A11 (ANXA11) were found. Importantly, several key membrane proteins specifically involved in tubular activity i.e., channels and membrane transporters were identified, such as the Solute carrier family 26 member 10 (S2610) and the Ammonium transporter Rh type C (RHCG).

### 3.4. Western Blotting of Differential uEV Proteins

Starting from the list of the identified proteins (Table 2), we selected some proteins and checked their differential uEV content using western blot analysis on single cases of each tubulopathy (4 for BS1, 4 for BS2, 5 for BS3 and 8 for GS patients). The choice of the proteins to be validated was guided by the following criteria: (i) identified only in one pool (Table 2); (ii) medium-high expression score in the kidney by Human Protein Atlas; (iii) implication in tubular transport processes. Using these criteria, we decided to evaluate uEV abundance of GIPC2, CA2, vacuolar-type ATPase B subunit 1 (VATB1), Na/H exchange regulatory cofactor NHE-RF1 (NHERF1) and RHCG. Moreover, we investigated the protein level of Annexin A2 (ANXA2), although it was not present in the gel spots, due to its involvement in the trafficking of several transmembrane channels or/and transporters at renal tubular level, such as NKCC2, the defective protein in BS1 [23]. The results are shown for GIPC2, CA2, VATB1, and ANXA2. NHERF1 and RHCG WB did not produce any useful data.

**Figure 4:**
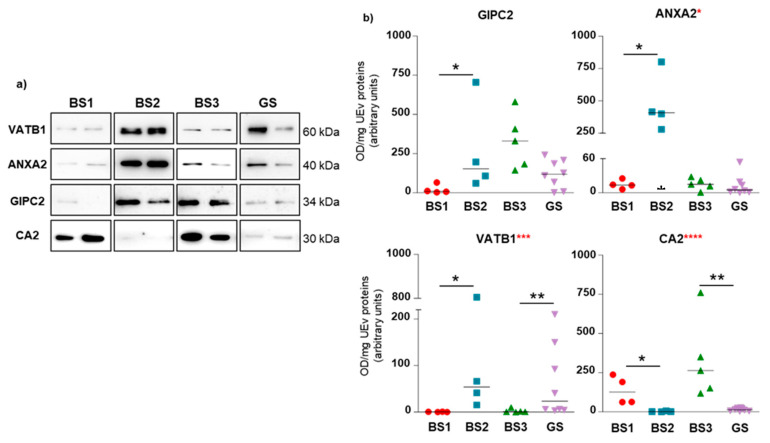
uEV content of the proteins of interest. (**a**) Immunoblotting of GIPC2, CA2, VATB1 and ANXA2 in uEV fraction of GS, BS1, BS2 and BS3 patients (two representative cases for each group are shown). Equal amounts of proteins were loaded on all lanes of each gel. (**b**) Densitometric analysis of protein signals normalized by mg of uEV proteins. Scatter plot of all patient data. Statistical analysis: red asterisks represent Kruskal-Wallis multivariate analysis, * *p*-value < 0.05; *** *p*-value < 0.001; **** *p*-value < 0.0001; black asterisks represent Mann-Whitney *t*-test: * *p*-value < 0.05; ** *p*-value < 0.01; *** *p*-value < 0.001.

The results of WB and densitometric analysis of the bands are summarized in Figure 4 (original western blotting images are provided in supplementary Figure S3). The data show that GIPC2 was found to be increased in BS2 uEV with respect to BS1; CA2 was more abundant in BS3 and BS1, compared to GS and BS2; also VATB1, being identified in 2 BS3 spots, had a differential level between the two couples BS1-BS2 and BS3-GS. Finally, ANXA2 was observed to be much more abundant in the uEV of BS2 cases with respect to those obtained from BS1. These findings suggest that the combined levels of the above proteins in uEV may allow the recognition of Gitelman syndrome and of the Bartter variants.

The issue of correctly normalising uEV protein content remains a hotly debated topic [21,22,23]. To normalize the uEV protein content we used both FLOT1, considered a “housekeeping” EV protein, and creatinine. The results of this further normalisation are shown in supplementary Figure S3 and agree with the reported data.

**Figure 5:**
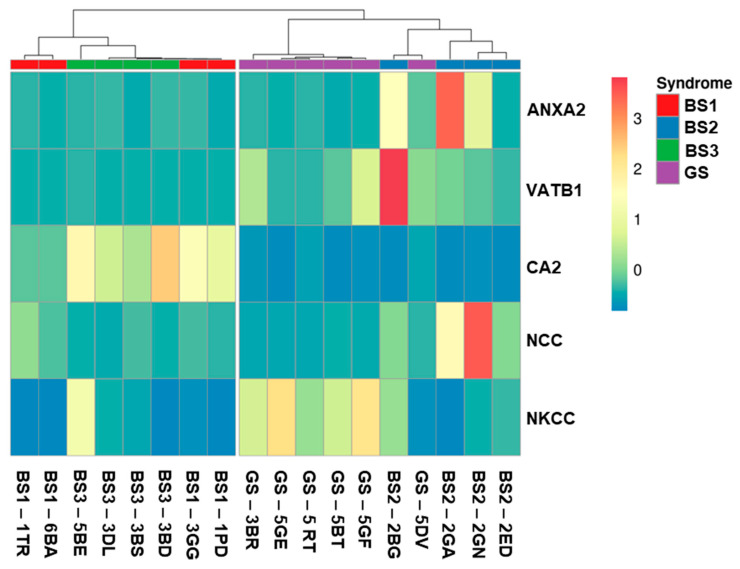
Expression pattern of the differential proteins. Heatmap representation of the band intensities (arbitrary units as in Figure 4) of the proteins of interest expressed as single values for each patient (https://biit.cs.ut.ee/clustvis/). We only included patients for whom we had the data for all the proteins considered.

After performing a multivariate analysis, we were able to provide an expression pattern as a heatmap representation (Figure 5). The best results were obtained including the relative uEV abundance of CA2, VATB1, ANXA2, along with NCC and NKCC2. For this reason, only patients for whom we had all these protein values were included in the heatmap. By using this protein panel, BS1 and BS3 cluster separately from GS and BS2, this allows a clear discrimination between BS1 and BS2 and between BS3 and GS.

## 4. Discussion

To our knowledge, this is the first study applying the 16-BAC/SDS-PAGE analysis to uEV. This choice is particularly suited to samples in which the ratio between membrane and soluble proteins is expected to be rather high due to the nanosize of the EV [26]. Actually, traditional 2-DE has many drawbacks in the separation of proteins with hydrophobic features [27,28]. In fact, they do not solubilize efficiently in the isoelectrofocusing (IEF) lysis buffer, which lacks strong ionic detergents, and can precipitate on the IPG strips, preventing them to be visualized on the second dimension gels. The diagonal bi-dimensional electrophoresis was proposed as a valid alternative. A recent publication, evaluating several protocols for the separation of plasma membrane protein (PMPs), reports that the number of identified PMPs (after density-gradient ultracentrifugation) was five-fold higher when 16-BAC/SDS-PAGE was used instead of 2DE [29]. In our case, the percentage of membrane proteins (plasma and organellar membranes) identified in the selected spots was 44%. Moreover, the integral transmembrane proteins represent 57% of the membrane proteins. Even if we analysed only a few spots, we believe that these results may be representative of all the uEV proteome. It has to be considered that, in general, the total coverage can never be assured for any proteome [30,31]. This is demonstrated by only partial overlapping of the identified proteins in several studies of the same models and as well as in uEV from healthy subjects, and these differences depend mainly upon the sample preparation and analysis protocols [24,25]. A recent study compared the uEV proteomes published in 4 different papers: each dataset contained between 1000 and 3500 identified proteins, yet only a minority of the proteins (238 proteins) were common to all studies [32].

It is noteworthy that several transporters and channels and their co-factors are among the identified proteins, such as AQP2, vacuolar-ATPases, Sodium/potassium-transporting ATPase, Solute carrier family 26 member 5 (Prestin) and S2610, Solute carrier family 12 member 3 (NCC), Sodium leak channel non-selective protein, NHERF1, Protein tweety homolog 3 (hTTY3). All these proteins are known to be enriched in renal cells, in particular in tubular epithelial types (www.proteinatlas.org). Some of them are involved in maintaining the body’s hydro-saline or acid base balance.

Our study focused on the comparisons between BS1 and BS2, and between GS and BS3, with the aim to provide a possible proteomic alternative to differentiate SLT. We chose and evaluated some proteins by western blot analysis and investigated whether they displayed differential abundances in uEV of the affected patients. These proteins are all involved in the transport of molecules across the plasma membrane of renal cells, as regulators of channel and/or transporter activity, or as direct modulators of acid-base balance.

CA2 is an enzyme located in the proximal tubule, loop of Henle, and intercalated cells in the collecting duct of the kidney [33]. It plays an important role in acid-base transport and salt reabsorption in the proximal convoluted tubule, where interplay among CA2, pendrin and cotransporter NCC was highlighted [34]. Moreover, mutations of CA2 gene per se cause mixed renal tubular acidosis, with osteopetrosis and mental retardation [35]. In addition, a very recent paper suggests that the use of Acetazolamide, a carbonic-anhydrase inhibitor, in combination with standard therapy in children with BS may counteract the metabolic alkalosis [36]. This report suggests a role for carbonic-anhydrase in the complex mechanisms underlying BS pathophysiology.

GIPC2 belongs to the GIPC family, proteins that contain a PDZ domain, functional to their specific interaction with transmembrane proteins [37]. Moreover, in pancreatic cancer cell lines GIPC regulates cellular trafficking pathways by modulating the secretion, biogenesis, and molecular composition of exosomes [38]. GIPC proteins were found concentrated in endocytic compartments of renal proximal tubule cells [39]. In particular, GIPC2 has a high expression protein score in kidney, especially in renal tubules (www.proteinatlas.org).

VATB1 represents the kidney isoform of the vacuolar-type ATPases [40] and is localized to the distal nephron [41]. It is involved in the intracellular pH regulation, by actively secreting protons into urine and heterozygous mutations have recently been reported in patients with some forms of renal tubular acidosis [42]. Furthermore, its alteration may cause neurosensory deafness, characteristic of Bartter variant 4 [43,44].

In relation to the annexin family, we identified two members (ANXA11 and ANXA7) in BS3 spots, but we investigated the uEV level of ANXA2, given that it has been demonstrated to co-distribute with NKCC2 to promote its apical translocation and surface expression [45].

The combined uEV levels of these proteins, together with the abundance of NCC and NKCC2 proteins, whose specific deficiency is the hallmark of GS and BS1, seem to discriminate among the four syndromes, and, in particular, between the two pairs of syndromes (GS/BS3, and BS1/BS2), which may present with overlapping clinical phenotypes. We are aware that the number of the patients is limited, but some of the syndromes are very rare. Moreover, we could rely on a well-characterized, although small, cohort where nearly all the mutations were identified and the clinical phenotypes were rather homogeneous. However, these results suggest that a specific uEV signature may characterize each condition.

Finally, it can be questioned whether the abundance of proteins in uEV reflect their actual level in the kidney. This requirement may be necessary if uEV are truly to serve as a “liquid biopsy.” This matter was discussed in a recent paper [46], demonstrating that this is not the case, at least for kidney-specific proteins and in healthy samples, and is further commented in [47]. As the authors suggest, the lack of correlation may derive from the method chosen by the authors to isolate uEV (precipitation with polyethylene glycol) combined with the high variability in the uEV abundance of the kidney-specific proteins often reported in healthy samples. It has to be underlined that authors normalised the loading amount of uEV to urinary creatinine, rather than normalising the expression of the various proteins to a marker of EV number (e.g., expression of CD63). In order to fully answer the question posed in their study they should have also looked at the relationship between protein expressions normalised to a marker of EV number. Moreover, the authors conclude that a relationship between kidney and uEV protein abundance may become apparent only in pathological samples because disease may upset normal regulation and cause a generalized and prolonged change in protein abundance that is reflected in uEV [39]. This may be exaggerated when the disease has a genetic origin, as in our case. Furthermore, the proteins with differential uEV contents among the syndromes may be involved in the putative mechanisms of adaptation/compensation that tubular cells implement in response to the primary genetic defect. This is especially true if we consider those proteins that modulate acid-base balance in relation to a common SLT symptom such as alkalosis. Of course, this small pilot study is not compelling enough to claim formal cause-to effect relationship, but it provides important proof of concept for the use of uEV profiles as a non-invasive tool for the initial rule out/in of suspect tubulopathies.

There is still much to learn about proteins that are involved in the expression and activity of the mutated transporters. Since there is still a subgroup of patients in whom no mutation can be found in the genes that are currently known to cause SLT, a deeper investigation of uEV proteome in these syndromes could lead to the discovery of dysregulated species and it could possibly generate targets for future treatments [2,6].

## 5. Conclusions

In conclusion, this paper highlights the role of the proteomic analysis on the uEV in tubulopathies suggesting the presence of a proteomic signature specific for each syndrome. In addition, the evaluation of the uEV content of a protein panel composed by the three selected potential biomarkers (CA2, VATB1 and ANXA2), added to NCC and NKCC2, provides proof of concept that differences in the proteome of uEV can be used to differentiate between different tubulopathies.

## Figures and Tables

**Table 1:** Genetic data of patients.

Patient Code	Age	Sex	Gene Affected	Mutation at DNA Level *	Changes at protein Level	Mutation Type	16-BAC	WB
**Gitelman Syndrome**								
3BR04	7	M	SLC12A3	c.[2981G>A]	p.[Cys994Tyr]	M		X
				c.[506-?_741+?del]	p.[Val169_Gln247del]	LD		
5BL94	16	M	SLC12A3	c.[2029G>A]	p.[Val677Met]	M	X	
				c.[506-?_741+?del]	p.[Val169_Gln247del]	LD		
5BT06	5	M	SLC12A3	c.[1924C>G]	p.[Arg642Gly]	M		X
				c.[2981G>A]	p.[Cys994Tyr]	M		
5CM97	14	M	SLC12A3	c.[2981G>A]	p.[Cys994Tyr]	M		X
				c.[2981G>A]	p.[Cys994Tyr]	M		
5CE94	17	F	SLC12A3	c.[1844C>T]	p.[Ser615Leu]	M	X	X
				c.[1925G>A]	p.[Arg642His]	M		
5DV85	26	M	SLC12A3	c.[2899A>G]	p.[Arg967Gly]	M	X	X
				c.[2899A>G]	p.[Arg967Gly]	M		
5GE01^	10	M	SLC12A3	c.[1196_1202dup7bp]	p.[Ser402*]	N		X
				c.[1424C>G]	p.[Ser475Cys]	M		
5GF01^	10	M	SLC12A3	c.[1196_1202dup7bp]	p.[Ser402*]	N		X
				c.[1424C>G]	p.[Ser475Cys]	M		
5MR86	25	F	SLC12A3	c.[2560delC];	p.[Leu854fs]	F	X	
				c.[1180+1G>T]	p.[Gly394fs]	F		
5RT94	18	M	SLC12A3	c.[238dupCC]	p.[Arg80fs]	F		X
				c.[2952-?_3090+?del]	p.[Ile984_Gln1030del]	LD		
**Bartter Type 1 Syndrome**								
1PD90	21	M	SLC12A1	c.[347G>A]	p.[Arg116His]	M	X	X
				c.[1954G>A]	p.[Gly652Ser]	M		
1TR02	9	M	SLC12A1	c.[551T>A]	p.[Leu184Gln]	M	X	X
				c.[611T>C]	p.[Val204Ala]	M		
3GG07	4	M	SLC12A1	c.[730dupG]	p.[Ala244fs]	F	X	X
				c.[1432G>A]	p.[Gly478Arg]	M		
6BA09	5	F	SLC12A1	c.[572T>A]	p.[Ile191Asn]	M	X	X
				c.[1493C>T]	p.[Ala498Val]	M		
**Bartter Type 2 Syndrome**								
2BG03	8	F	KCNJ1	c.[256A>G]	p.[Thr86Ala]	M		X
				c.[=]	p.[=]	-/-		
2ED04	3	M	KCNJ1	c.[133A>G]	p.[Lys45Glu]	M	X	X
				c.[?]	p.[Glu334fs]	F		
2GA04^	6	M	KCNJ1	c.[572C>T]	p.[Thr191Ile]	M	X	X
				c.[572C>T]	p.[Thr191Ile]	M		
2GN03^	7	M	KCNJ1	c.[572C>T]	p.[Thr191Ile]	M	X	X
				c.[572C>T]	p.[Thr191Ile]	M		
**Bartter Type 3 Syndrome**								
3BD82	29	M	CLCNKB	c.[371C>T]	p.[Pro124Leu]	M	X	X
				c.[371C>T]	p.[Pro124Leu]	M		
3BS07	4	F	CLCNKB	c.[725C>A]	p.[Ala242Glu]	M		X
				c.[1-?_2064+?del]	p.[0]	LD		
3DL91	20	F	CLCNKB	c.[1-?_2064+?del]	p.[0]	LD	X	X
				c.[1-?_2064+?del]	p.[0]	LD		
3SS82	29	M	CLCNKB	c.[1-?_2064+?del]	p.[0]	LD	X	X
				c.[1-?_2064+?del]	p.[0]	LD		
5BE99	12	F	CLCNKB	c.[1101G>A]	p.[Trp367*]	N	X	X
				c.[1?_2064+?del]	p.[0]	LD		

**Notes**: Patient code column: ^: indicates sibling; Unclassified = mutations in Gitelman and Bartter syndrome genes not identified. Mutation at DNA level column: *: genetic variants are shown for the two alleles; [=]: indicates undetected mutations; del: deletion; dup: duplication; [0]: putative null protein production because of complete deletion of the gene. Mutation Type column: M: missense mutation; N: nonsense mutation (stop); F: frameshift mutation; LD: large deletion. The samples used for 16-BAC/SDS-PAGE (16-BAC column) and for western blotting (WB column) analysis are marked with an “X” in the last two columns.

**Table 2:** List of the proteins identified in the differential spots of 16-BAC/SDS-PAGE of uEV pools of BS2 and BS3 patients.

Uniprot ID	Protein Name	Funct	Sub Loc	Mbr Inter	Kidney Expression Score
**Proteins Identified Only in BS2 Spots**					
P41181	Aquaporin-2	transp	mbr	M-P	medium
P08183	P-glycoprotein 1	transp	mbr	M-P	low
Q8IZF0	Na-leak channel non-selective protein *	transp	mbr	M-P	low
Q9NZM6	Polycystin-L2 *	transp	mbr	M-P	medium
Q9UBD6	Ammonium transporter Rh type C	transp	mbr	M-P	medium
P55017	Na-Cl cotransporter (NCC)	transp	mbr	M-P	medium
Q8NG04	Solute carrier family 26 member 10 *	transp	mbr	M-P	NA
P58743	Prestin *	transp	mbr	M-P	NA
Q9C0H2	Protein tweety homolog 3	transp	mbr	M-P	medium
Q9Y2B5	VPS9 domain-containing protein 1	transp	cyt		medium
P60709	Actin, cytoplasmic 1	struct	cyt		medium
Q9H6S3	EPS8-like protein 2	struct	cyt		high
Q96T17	MAP7 domain-containing protein 2 *	struct	nuc		low
Q96QZ7	Atrophin-1-interacting protein 3	struct	mbr	Per	medium
Q969L2	Protein MAL2	struct	mbr	M-P	low
Q92859	Neogenin	rec	mbr	S-P	low
P14384	Carboxypeptidase M	prot	mbr	L-A	high
P08246	Neutrophil elastase	prot	cyt		NA
P60900	Proteasome subunit α type-6	prot	cyt		medium
K7EIQ3	ZNF561 antisense gene protein 1 *	NA	NA		NA
Q9H579	Protein MROH8 *	NA	nuc		low
P0CG20	Proline-rich protein 35 *	NA	NA		NA
O94933	SLIT and NTRK-like protein 3 *	NA	mbr	S-P	NA
Q9Y4F4	Crescerin-1	NA	cyt		medium
O95336	6-phosphogluconolactonase	enz	cyt		high
O95573	Arachidonate-CoA ligase	enz	mbr	S-P	high
Q9BUT1	3-hydroxybutyrate DH type 2	enz	cyt		high
P00918	Carbonic anhydrase 2	enz	mbr	L-A	high
Q12873	ATP-dependent helicase CHD3	enz	nuc		low
Q13237	cGMP-dependent protein kinase 2	enz	mbr	L-A	medium
Q9BYJ1	Epidermis-type lipoxygenase 3 *	enz	cyt		NA
Q96RQ9	l-amino-acid oxidase *	enz	org		NA
Q8IY17	Neuropathy target esterase *	enz	mbr	S-P	high
P60174	Triosephosphate isomerase	enz	cyt		medium
P02768	Serum albumin	bind	secr		NA
P05090	Apolipoprotein D	bind	secr		high
P05026	Na/K-transporting ATPase sub β-1	bind	mbr	S-P	medium
Q16854	Deoxyguanosine kinase, mitochondrial *	bind	org		medium
Q0VDD8	Dynein heavy chain 14, axonemal *	bind	cyt		low
P01133	Pro-epidermal growth factor	bind	mbr	S-P	NA
O75955	Flotillin-1	bind	mbr	Per	high
Q86YZ3	Hornerin	bind	cyt		NA
Q8N1G4	Leu-rich repeat-containing protein 47	bind	cyt		high
Q8WV92	MIT domain-containing protein 1	bind	mbr	Per	high
P15941	Mucin-1	bind	mbr	S-P	medium
Q8WXI7	Mucin-16	bind	mbr	S-P	NA
P49321	Nuclear autoantigenic sperm protein	bind	cyt		low
O60422	One cut domain family member 3 *	bind	nuc		NA
Q9Y5G0	Protocadherin γ-B5	bind	mbr	S-P	NA
P05164	Myeloperoxidase	bind	org		NA
Q01970	Phospholipase C-β-3	bind	mbr	Per	low
P62191	26S protease regulatory sub 4	bind	cyt		nd
Q8NFJ5	Retinoic acid-induced protein 3	bind	mbr	M-P	low
P62263	40S ribosomal protein S14	bind	nuc		medium
P02743	Serum amyloid P-component	bind	secr		NA
P12931	Proto-oncogene c-Src	bind	mbr	Per	medium
Q16851	UDP-glucose pyrophosphorylase	bind	cyt		low
Q15904	V-type proton ATPase subunit S1	bind	mbr	S-P	high
Q9UN37	Vacuolar protein sorting-associated protein 4A	bind	mbr	Per	medium
O75351	Vacuolar protein sorting-associated protein 4B	bind	mbr	Per	medium
Q86YA3	protein ZGRF1 *	bind	mbr	S-P	medium
Q8IYB9	Zinc finger protein 595 *	bind	nuc		NA
B4DX44	Zinc finger protein 736 *	bind	nuc		medium
**Proteins Identified in Both BS2 and BS3 Spots**					
P53990	IST1 homolog (hIST1)	bind	cyt		high
P21281	V-ATPase subunit B 2	transp	mbr	Per	high
O43508	TNF ligand superfamily member 12 *	rec	mbr	S-P	medium
P01008	Antithrombin-III	prot inh	secr		medium
P01042	Kininogen-1	prot inh	secr		high
P16444	Dipeptidase 1	prot	mbr	L-A	high
P55786	Puromycin-sensitive aminopeptidase	prot	cyt		medium
Q9Y6R7	IgGFc-binding protein	na	secr		NA
P07948	Tyr-protein kinase Lyn	enz	mbr	Per	ND
P33908	Mannosidase α class 1A member 1	enz	mbr	S-P	high
P50995	Annexin A11	bind	cyt		medium
Q01518	Adenylyl cyclase-associated protein 1	bind	mbr	Per	medium
O75131	Copine-3	bind	nuc		low
P68104	Elongation factor 1-α 1	bind	cyt		medium
P02675	Fibrinogen β chain	bind	secr		NA
Q99816	Tumor susceptibility gene 101 protein	bind	mbr	Per	high
P0CG47	Polyubiquitin-B	bind	cyt		medium
P42685	FYN-related kinase	rec	cyt		medium
P19440	γ-glutamyltransferase 1	enz	mbr	S-P	high
**Proteins Identified Only in BS3 Spots**					
P01009	α-1-antitrypsin	prot inh	secr		NA
P01011	α-1-antichymotrypsin	prot inh	secr		low
P05154	Serpin A5	prot inh	secr		NA
P26038	Moesin	struct	mbr	Per	medium
Q8N957	ANKF1 *	na	nuc		NA
P04406	Glyceraldehyde-3-phosphate DH	enz	cyt		medium
P80723	Brain acid soluble protein 1	bind	mbr	L-A	ND
Q8TF65	PDZ domain-containing protein GIPC2	bind	cyt		high
P15313	V-ATPase subunit B 1	transp	mbr	Per	high
P04075	Fructose-bisphosphate aldolase A	struct	cyt		medium
P05062	Fructose-bisphosphate aldolase B	struct	cyt		high
Q9UHR4	BAI1-associated protein 2-like protein 1	struct	cyt		low
Q9UJU6	Drebrin-like protein	struct	cyt		medium
P15311	Ezrin	struct	mbr	Per	high
Q5S007	Leu-rich repeat Ser/Thr-protein kinase 2	struct	cyt		high
P22105	Tenascin-X	struct	secr		NA
P28223	Serotonin receptor 2A *	rec	mbr	M-P	NA
P46098	Serotonin receptor 3A *	rec	mbr	M-P	NA
P15328	Folate receptor α	rec	mbr	L-A	NA
Q9NQ84	Glycerol kinase	rec	mbr	M-P	high
Q08380	Galectin-3-binding protein	rec	secr		low
O14745	Na/H exchanger regulatory factor 1	rec	mbr	Per	high
Q6TCH4	Membrane progesterone P4 receptor δ *	rec	mbr	M-P	NA
O43653	Prostate stem cell antigen	rec	mbr	L-A	low
Q6UXT9	Protein ABHD15 *	prot inh	secr		medium
P05543	Thyroxine-binding globulin	prot inh	secr		NA
P28838	Cytosol aminopeptidase	prot	cyt		high
Q9Y646	Carboxypeptidase Q	prot	org		low
P81605	Dermcidin	prot	secr		NA
Q9UHL4	Dipeptidyl peptidase 2	prot	org		medium
Q6P179	Endoplasmic reticulum aminopeptidase 2	prot	mbr	S-P	medium
P12955	Xaa-Pro dipeptidase	prot	cyt		high
Q8IYX3	Coiled-coil domain-containing protein 116 *	na	cyt		NA
Q8NCU4	Coiled-coil domain-containing protein 191 *	na	org		medium
Q8WW52	Protein FAM151A	na	mbr	S-P	high
A8MUH7	PDZ domain-containing 1 pseudogene 1 *	na	NA		NA
Q9BW04	Specifically androgen-regulated gene protein	na	cyt		medium
P11766	Alcohol DH class-3	enz	cyt		high
P00352	Retinal DH 1	enz	cyt		low
P49419	α-aminoadipic semialdehyde DH	enz	cyt		high
Q9H2A2	Aldehyde DH family 8 member A1	enz	cyt		medium
P15121	Aldose reductase	enz	cyt		NA
Q92485	ASM-like phosphodiesterase 3b	enz	sec		NA
P16278	β-galactosidase	enz	org		medium
Q00796	Sorbitol DH	enz	mbr	Per	medium
Q9H9B1	Eu-HMTase1 *	enz	nuc		NA
Q6UWR7	GPC-Cpde ENPP6	enz	mbr	L-A	medium
P06744	Glucose-6-phosphate isomerase	enz	cyt		medium
P32189	Glycerol kinase	enz	mbr	Per	medium
P15309	Prostatic acid phosphatase	enz	mbr	S-P	NA
O43175	d-3-phosphoglycerate DH	enz	cyt		high
Q6PHR2	Ser/Thr-protein kinase ULK3	enz	cyt		high
Q96BJ3	AIDA protein	bind	cyt		low
P20073	Annexin A7	bind	nuc		medium
P06727	Apolipoprot A-IV	bind	secr		medium
P02649	Apolipoprot E	bind	secr		low
Q9UQB8	BAI-associated protein 2	bind	mbr	NA	high
Q9NRL2	BAZ protein 1A *	bind	nuc		low
Q8TDL5	BPI fold-containing family B member 1	bind	secr		NA
P55286	Cadherin-8	bind	mbr	S-P	NA
P08571	Monocyte differentiation antigen CD14	bind	mbr	L-A	NA
P10909	Clusterin	bind	secr		NA
Q14117	Dihydropyrimidinase	bind	cyt		high
Q96JB3	Hypermethylated in cancer 2 protein (Hic2) *	bind	nuc		low
P14618	Pyruvate kinase PKM	bind	cyt		medium
P30613	Pyruvate kinase PKLR	bind	cyt		high
Q96JY0	Protein maelstrom homolog *	bind	cyt		NA
Q86W25	Ntd-binding oligomerization domain protein 14 *	bind	NA		NA
Q14596	Next to BRCA1 gene 1 protein *	bind	cyt		medium
O94818	Nucleolar protein 4 *	bind	nuc		NA
Q6V1P9	Protocadherin-23 *	bind	mbr	S-P	NA
Q12913	Receptor-type Tyr-protein phosphatase η	bind	mbr	S-P	low
P35241	Radixin	bind	mbr	Per	high
Q9UPX8	Cortactin-binding protein 1	bind	mbr	NA	low
O95343	Homeobox protein SIX3 *	bind	nuc		NA
O60284	Suppression of tumorigenicity 18 protein *	bind	nuc	Per	high
Q9Y6E0	Ser/Thr-protein kinase 24	bind	cyt		medium
Q9BXI6	TBC1 domain family member 10A	bind	cyt		high
Q7Z2Z1	Treslin *	bind	nuc		NA
O75674	TOM1-like protein 1	bind	mbr	Per	medium
O60784	Target of Myb protein 1	bind	mbr	NA	medium
P59923	Zinc finger protein 445 *	bind	nuc		NA
Q92629	δ-sarcoglycan *	NA	mbr	S-P	NA
Q9Y6Q2	Stonin-1 *	NA	cyt		medium
Q8N1K5	Protein THEMIS *	NA	cyt		NA

Funct, function; Sub Loc, subcellular location; Mbr Int, membrane interaction type; Kidney expression Score, protein expression score in kidney, according to ProteinAtlas; NA, not available; ND, not detected; bind, binding protein; transp, transport protein; enz, enzyme; struct, structural protein; prot, protease; prot inh, protease inhibitor; rec, receptor; mbr, membrane; cyt, cytoplasm; org, organelle; nuc, nucleus; secr, secreted; M-P, multi-pass transmembrane protein; S-P, single-pass transmembrane protein; Per, peripheral membrane protein; L-A, lipid- anchor membrane protein; DH, dehydrogenase; Ser, Serine; Thr, Threonin; Tyr, Tyrosin; Leu, Leucin; Na, Sodium; K, Potassium; H, proton; BAI, Brain-specific angiogenesis inhibitor; TNF, Tumor necrosis factor; EPS8, Epidermal growth factor receptor pathway substrate; GPC-Cpde, Glycerophosphocholine cholinephosphodiesterase; ENPP6, Ectonucleotide pyrophosphatase/phosphodiesterase family member 6; ABHD15, Alpha/beta hydrolase domain-containing protein 15; MROH8, Maestro heat-like repeat-containing protein family member 8; ANKF1, Ankyrin repeat and fibronectin type-III domain-containing protein 1; ASM, Acid sphingomyelinase; Eu-HMTase1, Euchromatic histone-lysine *N*-methyltransferase 1; AIDA, Axin interactor dorsalization-associated protein; BAZ, Bromodomain adjacent to zinc finger domain. Proteins * indicate the 42 proteins identified in our data set and not in other uEV datasets [24,25].

## Supplementary Materials

The following are available online at https://www.mdpi.com/2227-7382/8/2/9/s1. Table S1: List of the proteins identified in the differential spots of 16-BAC/SDS-PAGE of uEV pool of Bartter Syndrome type 2 and 3 patients. Table S2: List of proteins identified in each analysed spot of BS2 uEV pool. Table S3: List of proteins identified in each analysed spot of BS3 uEV pool. Table S4: List of membrane proteins in our dataset. Figure S1: uEV characterization. Figure S2: Reproducibility of uEV protein profiles by 2D diagonal electrophoresis (biological and technical replicates). Figure S3: Maps of the spot identified in the diagonal electrophoresis gel of BS2 and BS3 uEV pools. Figure S4: Original western blotting images of the proteins of interest in all the analysed samples. Figure S5: Normalization of uEV protein content by FLOT1 and urinary creatinine.
