# Supplementary material for: Urinary Extracellular Vesicles and Salt-Losing Tubulopathies: A Proteomic Approach
Source: Proteomes. 2020 May 9;8(2):9. doi: 10.3390/proteomes8020009 (PMC7355747; doi:10.3390/proteomes8020009)
Supplement: Supplementary file 1 [file proteomes-08-00009-s001.zip › Supplementary/Figure_S1.pdf]

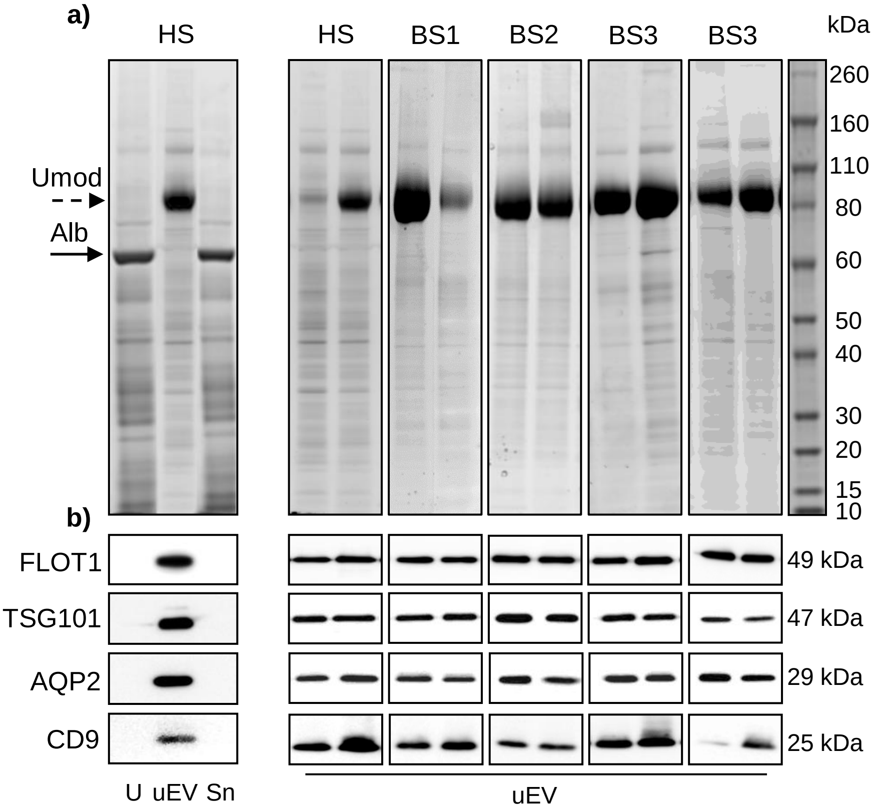


**Supplementary Figure S1. uEV characterization. a)** Protein profiles of uEV. NuPAGE 4-12% and Blue Coomassie staining of urinary extracellular vesicles (uEV) compared with total urine samples after sediment removal (U), and with supernatant (Sn) obtained after 200,000 xg ultracentrifugation from a representative healthy subject (HS); the dotted arrow indicates uromodulin (Umod) band typical of uEV; the continuous arrow indicates albumin (Alb) band enriched in U lanes (left panel). b) Reproducibility of UE protein profile: protein profiles of uEV isolated from HS, patients affected by Gitelman syndrome (GS), Bartter syndrome type 1 (BS1), type 2 (BS2) and type 3 (BS3); two representative cases for each group are shown (right panel). b) uEV marker enrichment. Immunoblotting for known uEV markers (FLOT1, TSG101, AQP2 and CD9) in uEV, U and Sn from a representative HS (left panel); markers enrichment in uEV from representative HS, GS, BS1, BS2 and BS3 patients (right panel). Equal amounts of proteins were loaded on all the lanes of each gel.
