# Supplementary material for: Urinary Extracellular Vesicles and Salt-Losing Tubulopathies: A Proteomic Approach
Source: Proteomes. 2020 May 9;8(2):9. doi: 10.3390/proteomes8020009 (PMC7355747; doi:10.3390/proteomes8020009)
Supplement: Supplementary file 1 [file proteomes-08-00009-s001.zip › Supplementary/Figure_S2.pdf]

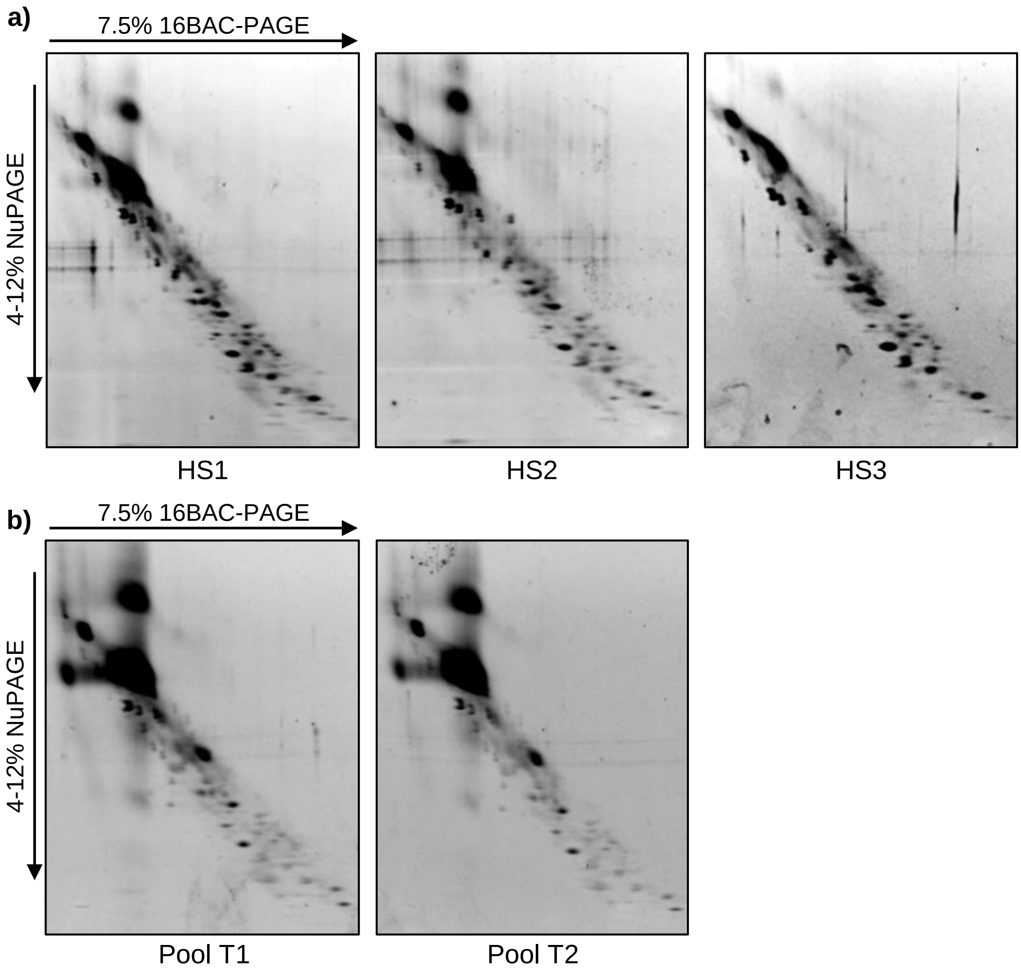

Supplementary Figure S2. Reproducibility of uEV protein profiles by 2D diagonal electrophoresis. a) Protein separation of uEV from three healthy subjects (HS1-3) by 16-BAC/SDS-PAGE and SyproRuby gel staining (biological replicates). b) Diagonal protein profile of the same uEV pool from BS3 patients analysed twice, at different times (T1 and T2) (technical replicates).
