# Supplementary material for: Urinary Extracellular Vesicles and Salt-Losing Tubulopathies: A Proteomic Approach
Source: Proteomes. 2020 May 9;8(2):9. doi: 10.3390/proteomes8020009 (PMC7355747; doi:10.3390/proteomes8020009)
Supplement: Supplementary file 1 [file proteomes-08-00009-s001.zip › Supplementary/Figure_S3.pdf]

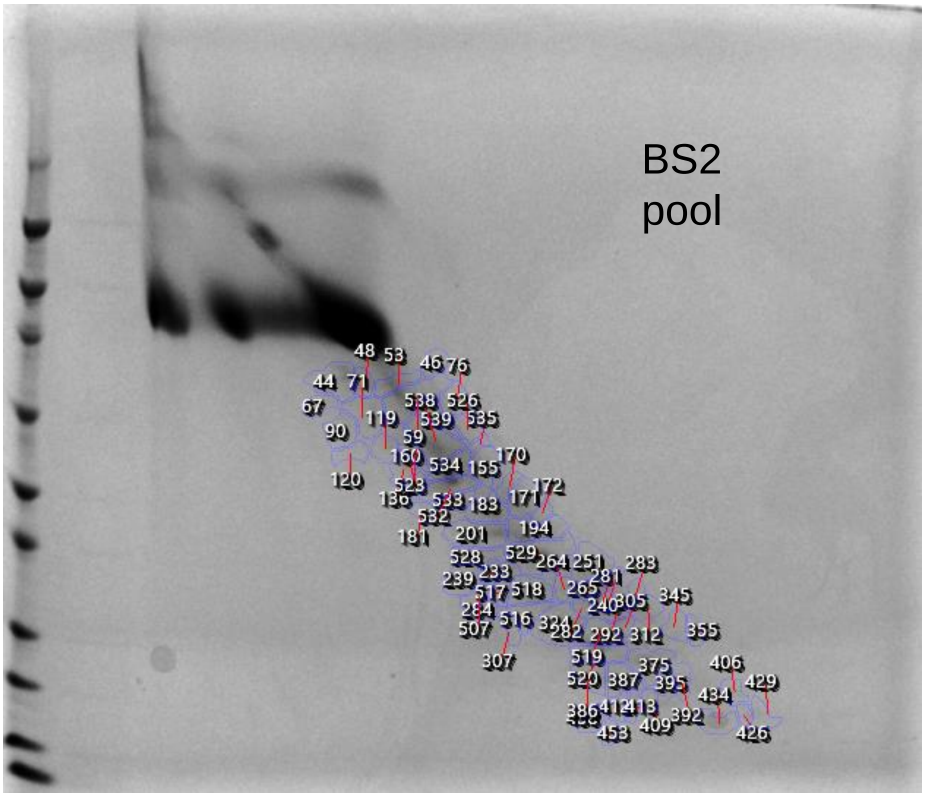

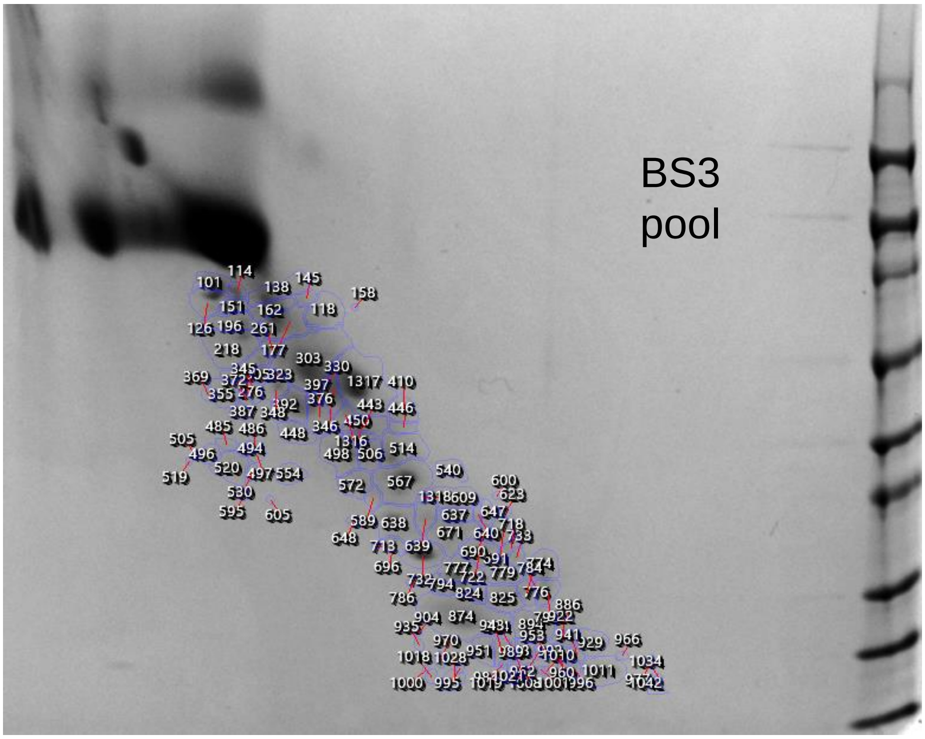

Supplementary figure S3. Number of spots obtained from image analysis (BS1 vs BS2 and BS3 vs GS) performed by SameSpots 2D Gel Image Analysis Software (TotalLab).
