# Supplementary material for: Urinary Extracellular Vesicles and Salt-Losing Tubulopathies: A Proteomic Approach
Source: Proteomes. 2020 May 9;8(2):9. doi: 10.3390/proteomes8020009 (PMC7355747; doi:10.3390/proteomes8020009)
Supplement: Supplementary file 1 [file proteomes-08-00009-s001.zip › Supplementary/Figure_S4.pdf]

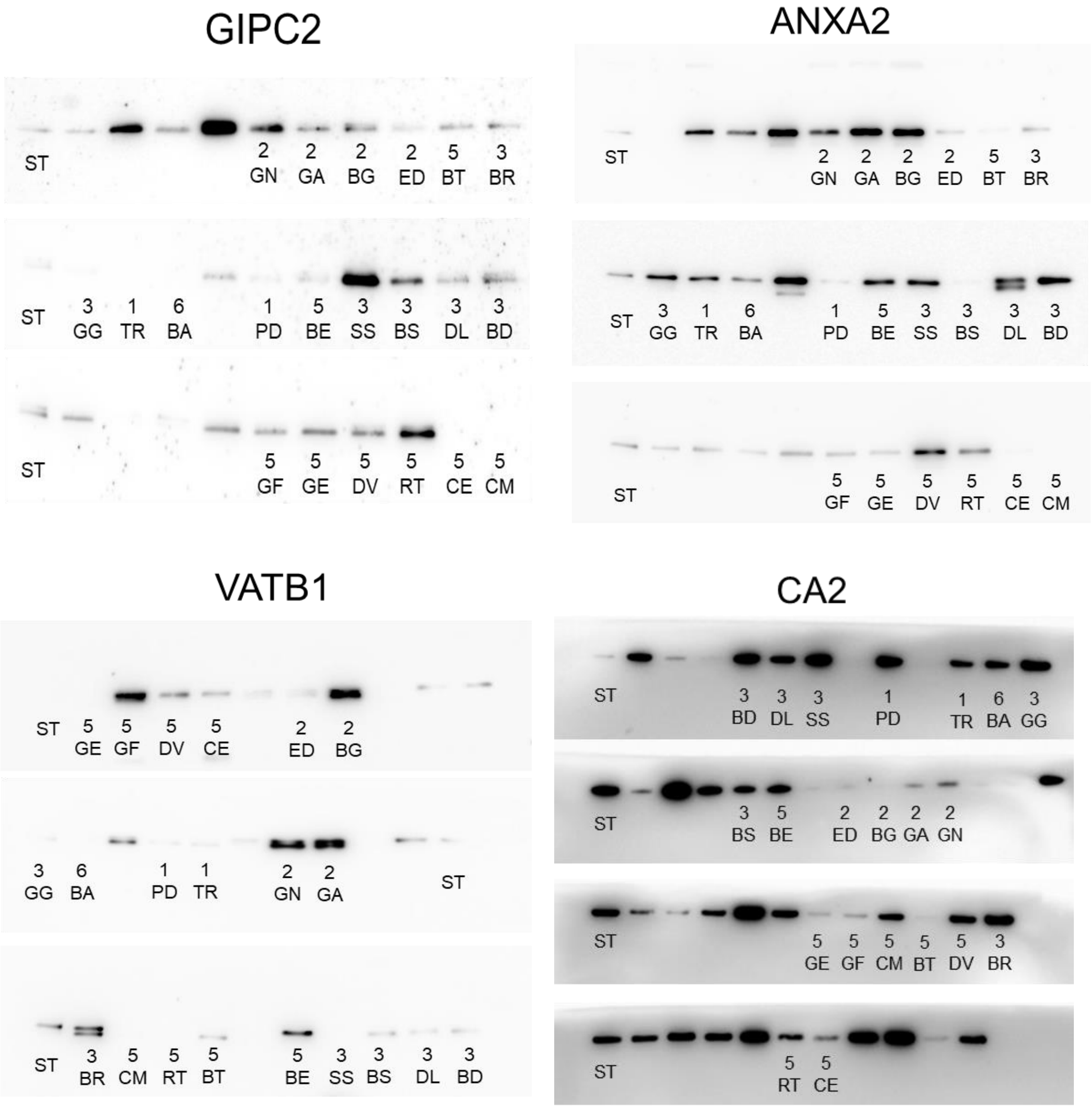

Supplementary Figure S4. uEV content of the proteins of interest. Original immunoblotting of GIPC2, CA2, VATB1 and ANXA2 in uEV fraction of GS, BS1, BS2 and BS3 patients. All the cases, whose data were discussed in the paper, are visible. Each patient is identified by the first 3 characters of his or her identification code. ST represent the same UE sample loaded in all gels: it was used as a correction factor for the signal detected from the different blots. The unnamed lanes represent uEV samples from control subjects and other types of kidney disease patients. Equal amounts of proteins were loaded on all lanes of each gel.
