# Supplementary material for: Urinary Extracellular Vesicles and Salt-Losing Tubulopathies: A Proteomic Approach
Source: Proteomes. 2020 May 9;8(2):9. doi: 10.3390/proteomes8020009 (PMC7355747; doi:10.3390/proteomes8020009)
Supplement: Supplementary file 1 [file proteomes-08-00009-s001.zip › Supplementary/Figure_S5.pdf]

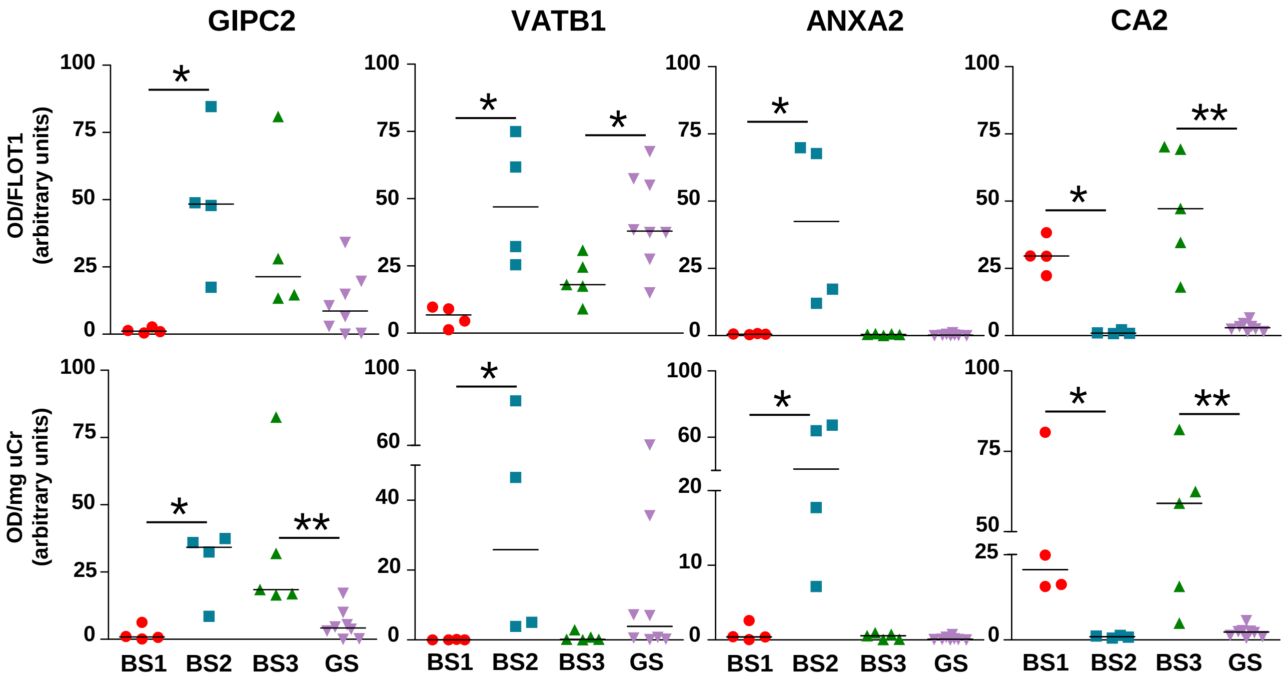

Supplementary Figure S5. Normalization of uEV protein content. Densitometric analysis of protein signals normalized by FLOT1, considered an EV "housekeeping" proteins (upper panels) and by urinany creatinine. Scatter plot of all patient data. Statistical analysis: red asterisks represent Kruskal-Wallis multivariate analysis, * *p*-value < 0.05; *** *p*-value < 0.001; **** *p*-value < 0.0001; black asterisks represent Mann–Whitney *t*-test: **p*-value < 0.05; ***p*-value < 0.01; ****p*-value < 0.001.
